# Exploring the Potential Application of an Innovative Post-Weld Finishing Method in Butt-Welded Joints of Stainless Steels and Aluminum Alloys

**DOI:** 10.3390/ma17081780

**Published:** 2024-04-12

**Authors:** Olga Łastowska, Robert Starosta, Monika Jabłońska, Andrzej Kubit

**Affiliations:** 1 Department of Engineering Sciences, Faculty of Marine Engineering, Gdynia Maritime University, 81-87 Morska St., 81-225 Gdynia, Poland; r.starosta@wm.umg.edu.pl; 2 Faculty of Mechanical and Electrical Engineering, Polish Naval Academy, 69 Jana Smidowicza Str., 81-127 Gdynia, Poland; m.jablonska@amw.gdynia.pl; 3 Department of Manufacturing and Production Engineering, Rzeszow University of Technology, al. Powst. Warszawy 8, 35-959 Rzeszow, Poland; akubit@prz.edu.pl

**Keywords:** post-weld finishing, stainless steel, aluminum alloy, surface quality, non-destructive testing, metallographic result, microhardness, microstructure, degree of hardening

## Abstract

The prerequisite of the weld bead finishing is intricately linked to the quality of the welded joint. It constitutes the final, yet pivotal, stage in its formation, significantly influencing the reliability of structural components and machines. This article delineates an innovative post-weld surface finishing method, distinguished by the movement of a specialized cutting tool along a butt weld. This method stands out due to its singular approach to machining allowance, wherein the weld bead height is considered and eradicated in a single pass of the cutting tool. Test samples were made of AISI 304L, AISI 316L stainless steels and EN AW-5058 H321, EN AW-7075 T651 aluminum alloys butt-welded with TIG methods. Following the welding process, the weld bead was finished in accordance with the innovative method to flush the bead and the base metal’s surface. For the quality control of welded joints before and after the weld finishing, two non-destructive testing methods were chosen: Penetrant Testing (PT) and Radiographic Testing (RT). This article provides results from the examination of 2D profile parameters and 3D stereometric characteristics of surface roughness using the optical method. Additionally, metallographic results are presented to assess changes in the microstructure, the microhardness, and the degree of hardening within the surface layer induced by the application of the innovative post-weld finishing method.

## 1. Introduction

In the assembly and fixation of steel structures and machine components, as well as during the installation of additional sealing, protection, or measuring elements, it is imperative to eliminate the entire weld seam allowance. In practice, a variety of techniques and tools are employed for the removal of weld beads. For example, methods of milling a welded joint are used. In a patent document [1], a method and a milling machine for machining ridge welds of roller elements that have been joined together are described. The method involves the movement of the ridge milling machine in a flat position, using chains, around the circumference of the connected cylindrical elements placed on a rotator. The milling cutter’s end mill removes the excess material from the ridge of the weld previously placed on the joint of the elements that have been joined together. Another very popular method of finishing welds is the grinding method [2,3]. In another patent document [4], a method for grinding external longitudinal welds and a device for grinding external longitudinal welds are described.

An essential objective is to devise a finishing technology for the weld that avoids compromising the operational characteristics of the welded joint, ensuring the reliable and durable performance of machine elements and connected structures. The innovative post-weld finishing method can be regarded as one such technology [5].

This solution involves finishing, achieved by moving a specialized cutting tool along the weld and removing the entire weld allowance in a single step [6,7]. Noteworthy features in the design of this tool include linearly arranged teeth with varying heights. The height of successive teeth incrementally changes in the direction opposite to the feed, and the disparity in blade height between the first and last teeth equals the height of the weld allowance [8,9]. This design results in a short processing time for the weld, leading to a substantial increase in the production efficiency of welded parts. Additionally, the designed tool ensures minimal deviations in shape and position, along with high accuracy and quality of the obtained weld bead surface layer [10].

The versatility of this solution enables the processing of products with varying shapes, constructed from different steels and alloys, utilizing a wide array of welding methods. The proposed post-welding finishing method holds potential for extensive applications across numerous industries such as ship repair, aerospace, armaments, rail transport, electric power, and others. It offers an alternative to the commonly used but inefficient, labor-intensive, and relatively expensive grinding method in various production processes. The grinding method is associated with the occurrence of defects on the treated surface. These defects encompass areas that are either missed or subjected to double hardening, resulting in structural notches in the surface layer. Additionally, residual stress, in the form of stretching, accumulates in these regions. These mentioned defects contribute to the initiation and propagation of both ordinary and fatigue cracks, as well as other forms of damage. During the operation of welded structures, the fatigue limit may be diminished, leading to the potential failure of welded elements. Moreover, grinding is a labor-intensive process often requiring manual work, significantly prolonging the time needed for finishing. Furthermore, grinding is detrimental to both human health and the environment, necessitating the implementation of additional protective measures.

We posit that, in contrast to conventional grinding and other contemporary weld finishing techniques, the innovative post-weld finishing method is not likely to manifest the drawbacks and inconveniences discussed earlier. Therefore, a comprehensive investigation into the impact of this innovative finishing method on the critical parameters governing weld surface layer quality and the strength of welded joints across various austenitic steels and aluminum alloys is imperative.

## 2. Materials and Methods

To investigate the viability of implementing the innovative post-weld finishing method, four material grades were selected. Among these, two widely utilized grades of austenitic stainless steels, AISI 304L and AISI 316L, were chosen for examination. Additionally, two grades of aluminum alloy, namely, EN AW-5083 H321 and EN AW-7075 T651, were included in the study. The contemporary scientific literature offers a wealth of captivating research concerning the welding of these selected materials. The investigation referenced as [11] delved into the influence of laser welding, on the mechanical properties and formability prediction of austenitic stainless steel AISI 304. The study on the microstructure, residual stresses, and stress corrosion cracking resulting from repair welding on 304 stainless steel is detailed in reference [12]. Article [13] aimed to evaluate the impact of different parameters in the tungsten inert gas (TIG) welding process on mechanical properties, stitch width, and microstructural characteristics of welds compared to 316 steel. Reference [14] discusses diverse butt joints between 6061-T6 aluminum alloy and AISI 316 stainless steel fabricated through friction stir welding (FSW) with varying parameters. The studies cited as [15] examined aluminum–magnesium (Al-Mg) alloys, particularly alloy AW 5083 and its joints using both MIG arc welding and FSW methods. Paper [16] explored parameter optimization for welding aluminum alloy AW 5083 via the synchropulse welding process. Article [17] focused on investigating potential correlations between mechanical properties, structural characteristics, and resistance to cavitation erosion in aluminum alloy 5083. Reference [18] details the examination of welded joints made from aluminum alloys EN AW-7075 and EN AW-6082, commonly used in car body construction, utilizing the low-energy cold metal transfer (CMT) arc welding method. Reference [19] presents a comparative analysis of welding EN AW 7075 aluminum alloy sheets using low-energy and pulsed current techniques. Finally, article [20] discusses the influence of semi-random and regular shot peening on selected surface layer properties of aluminum alloy. These materials find extensive applications across diverse industries, encompassing modern marine [15,21] and mechanical engineering [22,23], the military [24], chemical, aerospace, automotive [18], rail [25], and other [26]. Thus, it is imperative to explore the feasibility of an innovative post-weld finishing method for butt-welded joints involving the aforementioned materials.

Stainless steel 304L is a standard grade within the austenitic chromium–nickel steels group. It exhibits good corrosion resistance, primarily in natural environments; however, it is not suitable for use in saline environments and environments with high chlorine concentrations. At elevated temperatures, such as during welding, there is a risk of intergranular corrosion. In contrast, AISI 316L steel is an acid-resistant austenitic stainless steel. Its superior corrosion resistance, attributed to the presence of molybdenum, sets it apart from AISI 304L. Steel 316L demonstrates resistance to both organic and inorganic acids as well as chloride-containing agents. Moreover, it is less prone to pitting corrosion. The low carbon content further enhances its resistance to intergranular corrosion, enabling its utilization at elevated temperatures.

The chemical composition and mechanical properties of these material, as per the inspection certificate EN 10204-3.1 (mill test certificate), are detailed in Table 1 and Table 2.

Another group of materials under investigation includes aluminum alloys of EN AW-5083 H321 and EN AW-7075 T651 grades. The key advantages of aluminum alloy EN AW-5083 H321 encompass excellent corrosion resistance, superb chemical resistance, and resistance to seawater. This grade exhibits moderate strength with optimal resistance to hardening. Notably, due to its chemical composition, aluminum EN AW-5083 H321 is characterized by good deformability. Interestingly, while not recommended for machining, it excels in welding applications owing to its anti-corrosion properties (attributed to its low copper content) and high fatigue strength. The aluminum alloy EN AW-7075 T651 belongs to the 7000 series, specifically zinc alloys. These alloys typically consist of 5–8% zinc and approximately 2% magnesium, with possible additional elements such as copper, chromium, or titanium. Recognized as the strongest aluminum alloys, they boast tensile strengths of up to 600 MPa, comparable to structural steels, and exhibit an exceptionally high fatigue strength. The addition of copper to these alloys reduces susceptibility and enhances achievable strength. However, copper-containing alloys may face drawbacks such as reduced resistance to atmospheric corrosion and increased susceptibility to notching. Despite being an excellent material for machining, EN AW-7075 T651 is unsuitable for welding and possesses less corrosion resistance compared to many other alloys.

The chemical composition of EN AW-5083 H321 and EN AW-7075 T651 aluminum alloys according to the inspection certificate EN 10204-3.1 is provided in the data sheet below (Table 3).

The mechanical properties of EN AW-5083 H321 and EN AW-7075 T651 are outlined in Table 4.

**Figure 1:**
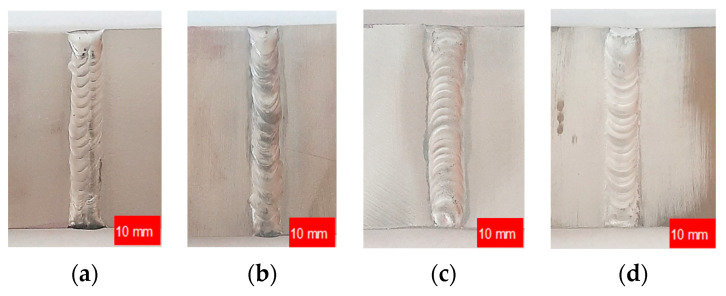
Samples of butt-welded joints fabricated from the chosen materials: (**a**) AISI 304L I_w_ = 140 A; (**b**) AISI 316L I_w_ = 140 A; (**c**) EN AW-5083 H321 I_w_ = 160 A; (**d**) EN AW-7075 T651 I_w_ = 170 A.

Following material selection, test specimens measuring 200 mm in length, 70 mm in width, and 6 mm in thickness were fabricated. These specimens comprised single V-shaped butt-welded joints using TIG at a specified current strength (I_w_), as depicted in Figure 1.

In the subsequent stage of the investigation, the samples underwent two distinct non-destructive testing (NDT) methods: Penetrant Testing (PT) and Radiographic Testing (RT) to identify welding defects. These techniques are commonly employed in the examination of welded joints. For instance, in article [27], the author meticulously analyzes specific non-destructive testing (NDT) methodologies, highlighting their significance in assessing welded joints utilized in maritime transportation structures. Additionally, reference [28] explores contemporary non-destructive testing methodologies for welded joints. The objective outlined in paper [29] is to assess the prevailing standards and methodologies for predicting the fatigue life of welded joints in the marine industry. This study critically evaluates various approaches, elucidating their respective advantages and limitations. In paper [30], a study on non-destructive testing is presented, utilizing micro-focused X-ray tomography to comprehensively evaluate welds, including internal defects such as cracks and pores. Furthermore, paper [31] employs X-ray CT to analyze the progression of damage in extrusion welds nearing failure.

**Figure 2:**
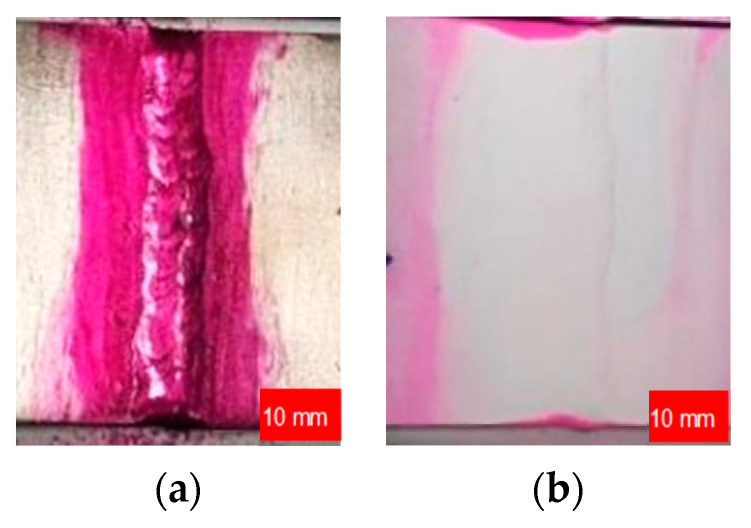
The progress of the Penetrant Test on the butt-welded joint: (**a**) suspension penetrant agent; (**b**) developer.

Penetrant inspection was conducted before and after the samples’ finishing in accordance with PN-EN ISO 3452-1 and PN-EN ISO 23277, utilizing equipment such as illumination LX-105 (Lutron Electronics Co., Inc., New York City, NY, USA) and pyrometer CHY110 (CHY Firemate Co., Ltd., Tainan, Taiwan). The penetrant agent used was suspension, with developer, and remover solvent. Test parameters included the use of indicator, the spray method, natural drying, a penetration time of 25 min, a developing time of 25 min, and a temperature of the object and ambient temperature set at 15 °C. The progress of the study is illustrated in Figure 2.

**Figure 3:**
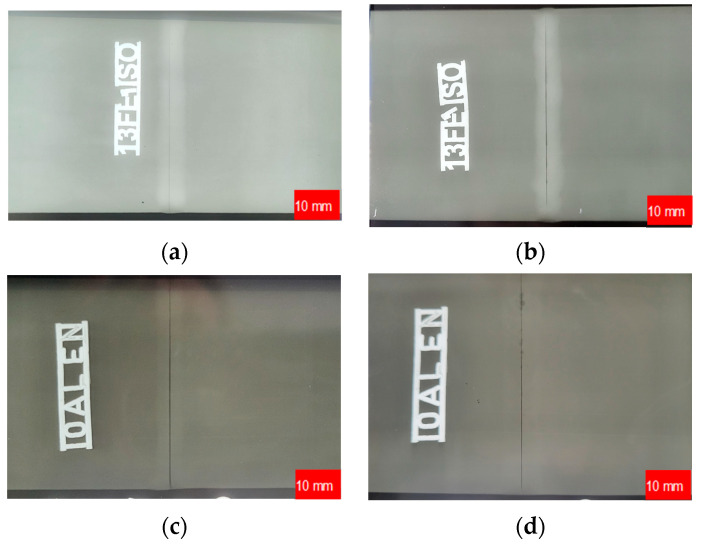
Radiographic testing of a butt-welded joint: (**a**) AISI 304L; (**b**) AISI 316L; (**c**) EN AW-5083 H321; (**d**) EN AW-7075 T651.

Radiographic testing was conducted using X-ray techniques with film in compliance with PN-EN ISO 17636-1 and PN-EN ISO 6520-1. The test equipment included an X-ray source with a membrane system of C3/D4 class, film type 3 × 3 mm, densitometer Densorapid D (KOWOTEST GmbH, Langenfeld, Germany), developing machine, film illuminator Kowolux X3 Led (KOWOTEST GmbH, Langenfeld, Germany), and RTG lamp YXLON YPO EVO 2250 (Yxlon GmbH, Hamburg, Germany). The progress of the study is depicted in Figure 3.

**Figure 4:**
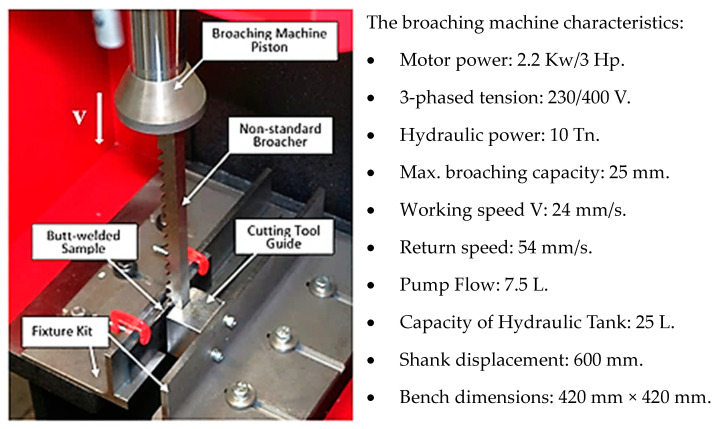
The test stand based on the hydraulic broaching machine.

Subsequently, the weld bead of the samples was removed using the innovative post-weld surface finishing method [5,6,7]. To implement this distinctive method, a non-standard multi-tooth cutting tool (broacher) was conceptualized and manufactured [32], along with a fixture kit designed for the installation, positioning, and securement of both the cutting tool and the welded specimen [8,9]. The engineered set, constructed from steel, was firmly affixed to the stationary tabletop of the hydraulic broaching machine BM25 (PRADA NARGESA S.L., Girona, Spain). The kit comprised three structural components: left and right angles made of unequal-sided steel and a stainless-steel cutting tool guide (Figure 4) [10].

**Figure 5:**
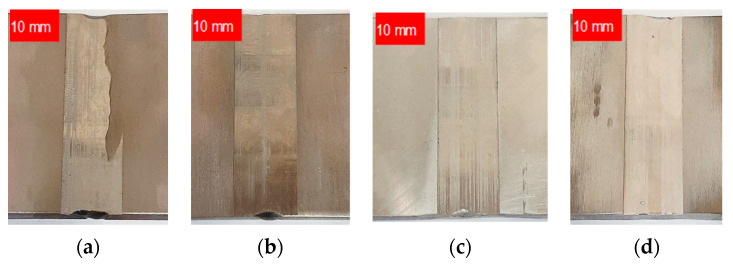
Samples of butt-welded joints after finishing: (**a**) AISI 304L; (**b**) AISI 316L; (**c**) EN AW-5083 H321; (**d**) EN AW-7075 T651.

This method facilitates an accurate and a high-quality removal of weld beads [5]. Figure 5 shows welded specimens after finishing.

To visually analyze the obtained surface and identify areas of characteristic roughness, images were captured at a magnification of 32 times using a SmartZoom 5 digital industrial microscope by Zeiss (Carl Zeiss AG, Oberkochen, Germany) [5].

After visually analyzing them, to investigate the impact of the innovative method for removing excess material from welds in specimens made of various stainless steels and aluminum alloys on the key parameters of the surface geometric structures [33,34], surface roughness measurements were conducted using a non-contact optical method extensively described in the modern scientific literature [35,36].

Additionally, a visual representation of the surface roughness and topography [37] of the selected area was obtained using the Alicona Infinite Focus G6 (Alicona Imaging GmbH, Vienna, Austria). Both 2D profile parameters (Ra, Rz, Rt, Rv, Rz) and stereometric characteristics of the 3D surface roughness (Sq, Sp, Sv, Sz, Sa), determining its functional properties, were examined [38].

The Abbott-Firestone curve, or bearing area curve (BAC), characteristics were also evaluated. The curve describes the surface texture of an object and can be obtained from a profile trace by drawing lines parallel to the datum and measuring the fraction of the line that lies within the profile. Mathematically, it is the cumulative probability density function of the surface profile’s height and is calculated by integrating the probability density function.

In the next stage of the study, metallographic analysis [17,39] was conducted to assess changes in the microstructure [40,41], microhardness [42], and degree of hardening within the surface layer [43] induced by the application of the innovative post-weld finishing method.

Samples for testing were prepared using standard methods in accordance with the assistance of special equipment (sample cutter, automatic grinder-polisher, sample inlay press). Additionally, reference specimens, taken immediately after the welding process, were selected for testing. Microstructural observations were conducted in the etched state with Marble/No. 25 reagents according to ASTM E407 (for steel) and Keller/No. 4 according to ASTM E407 (for aluminum alloys). Metallographic specimens were observed using light microscopy (LM) with a Leica DMI-3000X (Leica Microsystems GmbH, Wetzlar, Germany) metallographic microscope and scanning electron microscopy (SEM) with a Hitachi S-3400N (Hitachi High-Tech Corporation, Tokyo, Japan) scanning electron microscope. A backscattered electron (BSE) detector was utilized during SEM studies, with a pinning voltage set at 20 kV.

Microhardness measurements were conducted using the Vickers method with a NEXUS 4303 (INNOVATEST Europe BV, Maastricht, The Netherlands) micro-hardness tester, following ASTM E384 standards. A load of 0.25 N (25G) was applied to aluminum alloys, while 0.5 N (50G) was applied to steel. The degree of hardening (U) of the weld surface was determined as the relative percentage increase in hardness using the following relation:

$$U=\frac{{HV}_{p}\cdot {HV}_{s}}{{HV}_{s}}\cdot 100\%,$$

where *HV*_p_—the hardness of the weld after finishing; *HV_s_*—the hardness of weld bead before finishing.

## 3. Results and Discussion

Non-destructive testing, including penetrant and radiographic examinations of welded joints prior to finishing, did not reveal significant welding inconsistencies. The only welding defect to be considered is the occurrence of pores at the ends of the weld in the test specimens, likely resulting from the absence of run-out plates in the joints. Considering this factor, and given that only a relatively small part of the weld bead (approximately 30% of the entire length) from the middle part will be cut out for further tests, the mentioned defect can be deemed minor, and the test results are accepted with allowable indications.

**Figure 6:**
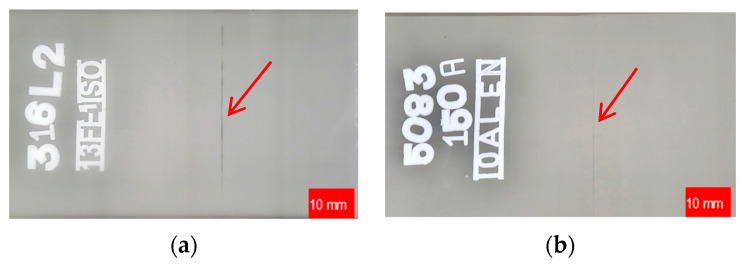
RT inspection of a butt weld joint after finishing: (**a**) AISI 316L; (**b**) EN AW-5083 H321; arrows indicate the lack of penetration of the weld.

The results of the non-destructive testing conducted after the removal of the weld bead according to the innovative method were not entirely conclusive. RT inspection showed no welding defects in the test specimens (Figure 6).

**Figure 7:**
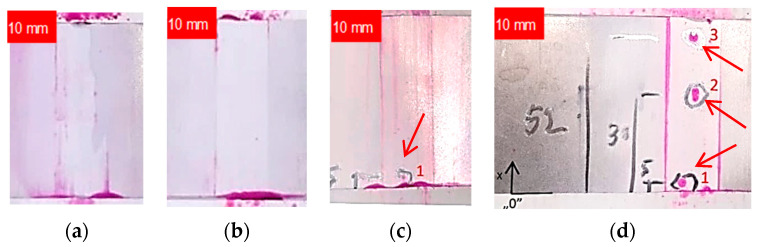
Penetrant inspection of welded joint samples after finishing: (**a**) AISI 304L; (**b**) AISI 316L; (**c**) EN AW-5083 H321; (**d**) EN AW-7075 T651; arrows depict the locations of the defects.

Nevertheless, the results of the Penetrant Testing on welded stainless-steel specimens subjected to finishing did not reveal any defects related to the welding process or finishing of the weld bead. However, the PT inspection of aluminum alloys unveiled defects on the finished weld bead surface that are deemed unacceptable. Figure 7 illustrates the tested samples, providing a detailed depiction of the visible defects. Table 5 offers a description and specifies the locations of the indications.

The penetration tests demonstrate that the application of an innovative method for removing weld bead allows for the detection (open) of weld inconsistencies, enabling their repair before the welded component is put into operation. This can contribute to enhancing the reliability of welded structures.

**Figure 8:**
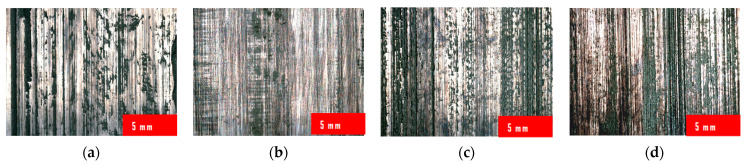
Weld bead surface after finishing at a magnification of 34 times: (**a**) AISI 304L; (**b**) AISI 316L; (**c**) EN AW-5083 H321; (**d**) EN AW-7075 T651.

The results of the visual analysis of the weld bead surface finishing according to the innovative method at a magnification of 34 times are shown in Figure 8.

The results of the investigation into the influence of the innovative post-weld finishing method on specimens made of various stainless steels and aluminum alloys on 2D profile parameters are presented in Table 6.

The impact of the amplitude parameters of surface roughness, such as Ra (Rz) and Rt, on fatigue strength was found to be crucial. This was attributed to the fact that the depth of the profile grooves served as an indicator of stress concentration. Comparing the results of all the welded specimens machined according to the innovative method, it was found that the lowest roughness value of Ra (Rz) = 0.295 (2.560) μm was obtained when measuring the weld-joint made of 316L stainless steel. Conversely, the highest Ra (Rz) = 1.636 (9.268) μm was measured on a sample made of EN AW-5083 H321 aluminum alloy.

The butt welds that underwent finishing were also assessed based on surface topography (Table 7). The following parameters were analyzed: the root-mean-square height, or the root-mean-square deviation of the surface Sq, which was defined analogously to Rq and calculated as the standard deviation of the height of surface irregularities with respect to the reference surface; the height of the highest surface elevation Sp (maximum peak height); the depth of the lowest pit Sv (maximum pit depth); the maximum surface height—formerly the height of the surface profile ordinates from 10 points Sz (maximum height); the arithmetic mean surface height Sa (arithmetic mean height).

The largest value of the Sv parameter, equal to 41.89 μm, was obtained when machining welds made of aluminum alloy EN AW-7075 T651. The smallest value of the maximum pit depth indentation was achieved by implementing finishing for a weld-joint made of austenitic steel AISI 316L. For AISI 304L stainless steel, the highest value of Sp = 25.65 μm was observed, significantly deviating from the values of this parameter measured for other materials. This discrepancy may be attributed to the transfer of the overgrowth material fragment to the machined surface. The weld finishing, conducted according to the innovative method, allowed for the attainment of a surface roughness characterized by low values of the Sa parameter, ranging from 1.32 μm to 1.64 μm. Such relatively low values of the Sa parameter are typical of finishing operations. Regarding Sz values, the obtained range was from 23.22 μm to 47.83 μm, but these values may also occur after grinding or polishing operations.

**Figure 9:**
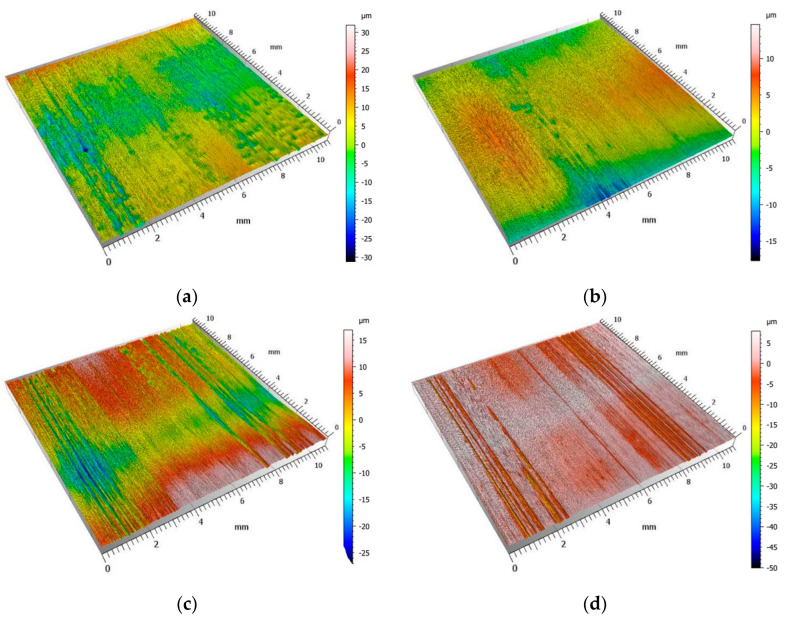
Isometric images of weld-joint surfaces after finishing: (**a**) AISI 304L; (**b**) AISI 316L; (**c**) EN AW-5083 H321; (**d**) EN AW-7075 T651.

Figure 9 shows isometric images of weld surfaces after finishing.

Valleys, defined as long depressions of surface irregularities, were observed on all surfaces. The crack formation on the machined surface is caused by the uneven cut depth in the cross-section. This unevenness is caused by overgrowths formed on the cutting teeth edge. During the cutting of materials characterized by high plasticity, locally on the rake surface near the cutting tool’s edge, overgrowths form from the crush-reinforced particles of the workpiece material. Due to its much higher hardness, the overgrowth represents an “extension” of the cutting edge and is often involved in the cutting of the plastic weld material. The resulting overgrowth influences the shape of the cutting edge and affects the cutting forces and, locally, the depth of cut. As a result of the resulting overgrowth, the cutting surfaces are characterized by the occurrence of valleys arranged in the direction of cutting. The occurrence of deep valleys can also be indicated by the values of the Sv parameter (Table 7).

**Figure 10:**
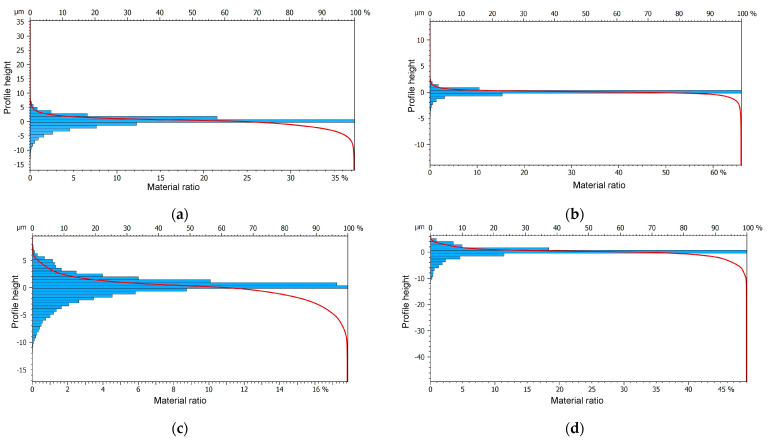
The bearing area curves of weld-joint surfaces after finishing: (**a**) AISI 304L; (**b**) AISI 316L; (**c**) EN AW-5083 H321; (**d**) EN W-7075 T651.

The analysis of the bearing area curve (Abbott-Firestone curve) shown in Figure 10 confirms the aforementioned statements.

The purpose of the metallographic study was to evaluate changes in microstructure and hardness in the near-surface layer in the welded joint area caused by the surface machining process. In accordance with the research methodology, the following procedures were conducted:

1. An evaluation of the alteration in the microstructure within the zone of plastic deformation of the material after surface treatment, along with the determination of the depth of its occurrence,
2. A determination of the variation in microhardness of the weld from the surface into the depth of the weld,
3. An assessment of the degree of hardening subsequent to the machining of the plastic deformed layer.

**Figure 11:**
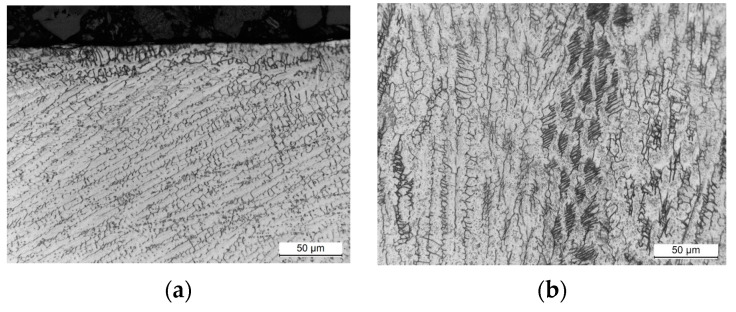

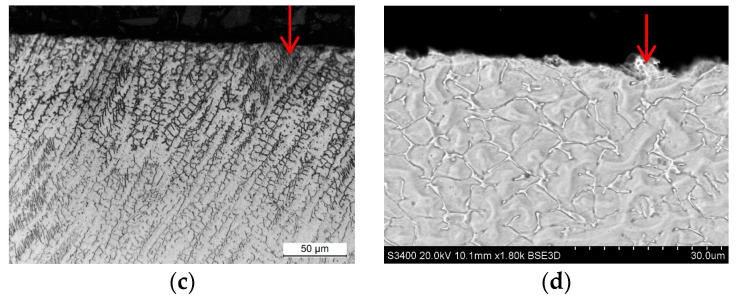
Weld microstructure of AISI 304L steel: (**a**) before machining in the near-surface area with a 500× magnification (LM), (**b**) before machining inside the section with a 500× magnification (LM), (**c**) after finishing in the near-surface area with a 500× magnification (LM), (**d**) after finishing in the near-surface area with a 1800× magnification (SEM); arrows indicate the plastically deformed zone.

**Figure 12:**
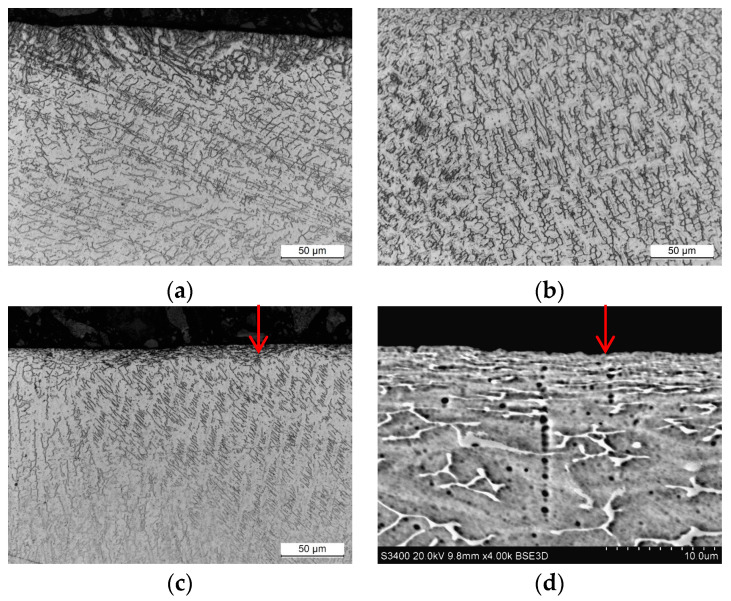
Weld microstructure of AISI 316L steel: (**a**) before machining in the near-surface area with a 500× magnification (LM), (**b**) before machining inside the section with a 500× magnification (LM), (**c**) after finishing in the near-surface area with a 500× magnification (LM), (**d**) after finishing in the near-surface area with a 4000× magnification (SEM); arrows indicate the plastically deformed zone.

**Figure 13:**
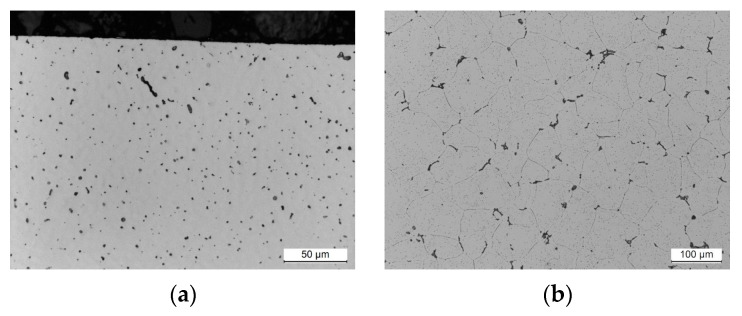

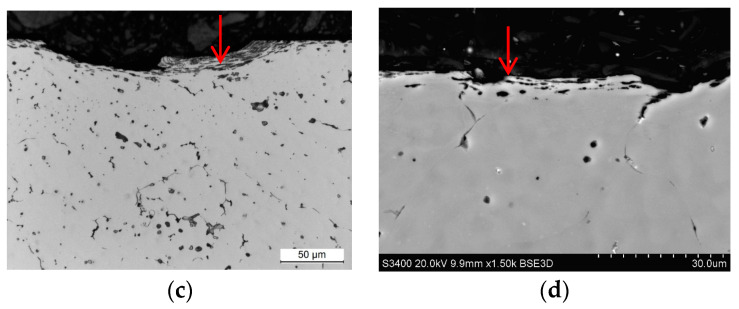
Weld microstructure of aluminum alloy EN AW-5083 H32: (**a**) before machining in the near-surface area with a 500× magnification (LM), (**b**) before machining inside the section with a 200× magnification (LM), (**c**) after finishing in the near-surface area with a 500× magnification (LM), (**d**) after finishing in the near-surface area with a 1500× magnification (SEM); arrows indicate the plastically deformed zone.

**Figure 14:**
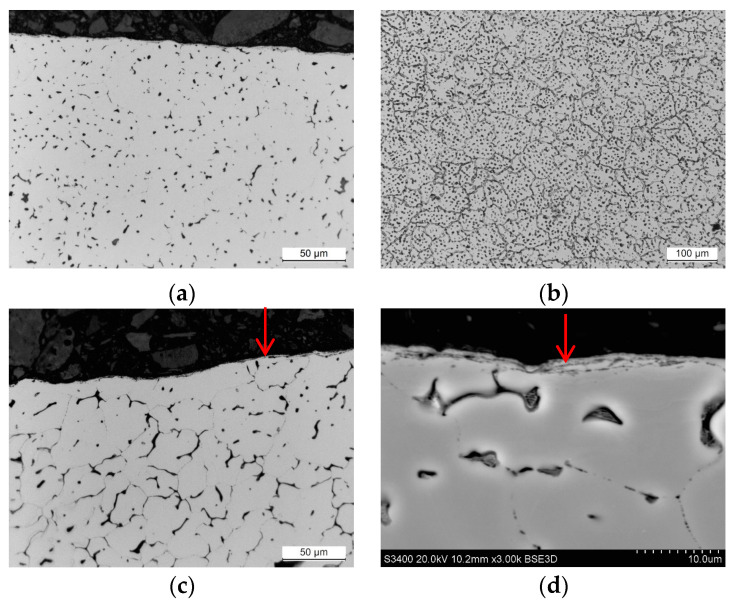
Weld microstructure of aluminum alloy ENAW-7075 T65: (**a**) before machining in the near-surface area with a 500× magnification (LM), (**b**) before machining inside the section with a 200× magnification (LM), (**c**) after finishing in the near-surface area with a 500× magnification (LM), (**d**) after finishing in the near-surface area with a 3000× magnification (SEM); arrows indicate the plastically deformed zone.

Microstructural observations were conducted on samples obtained from weld cross sections. In the near-surface regions of all observed specimens, a layer characterized by alterations in the microstructure was identified. This layer formed as a consequence of surface finishing, involving plastic deformation of the material. The results of the study are illustrated in Figure 11, Figure 12, Figure 13 and Figure 14.

Studies of the microstructure carried out on metallographic specimens taken from weld cross-sections revealed the presence of a zone characterized by alterations in the microstructure in areas adjacent to the physical surface of the machined materials. These changes resulted from the applied finishing operations. The alterations identified in this zone are linked to the plastic deformation of the material induced by the occurrence of sufficiently high cutting forces. The magnitude of these forces exceeded the yield strength of the machined material. It was found that for both grades of stainless steel, the flow lines were clearly visible while the deformation of the near-surface zone in the aluminum alloys was much more difficult to observe.

The depth of the deformed zone and the concentration of deformation lines varied depending on the material of the samples. The width of the plastic deformation zone after the applied surface finishing was determined by directly measuring the depth of the microstructural changes in the plastically deformed zone. For each specimen, measurements were made in five distant areas, initially determining the thickness of the zone at a point. The arithmetic mean value of the 15 thickness values determined at the point was taken as the average thickness of the plastically deformed zone (Table 8).

Measurements of the plastically deformed zone indicated that welds of steel material underwent greater plastic deformation than welds of aluminum alloys. The weld material of 316L machined steel deformed to a depth of about 6 µm, while the microstructure of the EN AW-7075 T651 aluminum alloy changed over an area of only 2.3 µm from the surface of the actual weld. The depth of the plastically deformed area is attributed to the occurrence of different values of cutting forces during machining process. Higher cutting forces were experienced during the finishing of stainless-steel welds than during the machining of softer welds of aluminum alloys.

**Figure 15:**
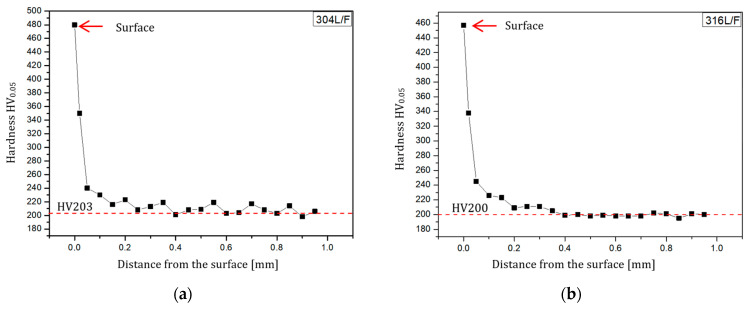

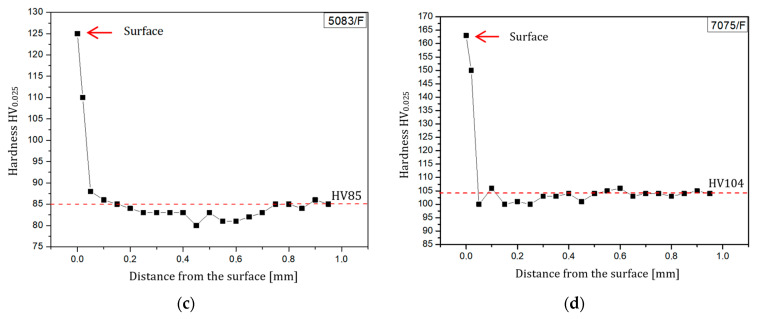
Curves of microhardness dependence on the distance from the weld surface: (**a**) AISI 304L; (**b**) AISI 316L; (**c**) EN AW-5083 H321; (**d**) EN AW-7075 T651.

Hardness measurements, similar to microstructural observations, were conducted on specimens obtained from weld cross-sections. Measurements were taken from the surface in a direction perpendicular to the weld surface. The surface hardness in the weld area was determined by measurements directly on the surface. The results of hardness measurements are presented in Figure 15 in the form of curves depicting the dependence of weld microhardness on the distance from the weld surface.

The microhardness measurements confirmed the findings from the microstructural observations. Changes resulting from post-weld finishing were more pronounced in steel specimens compared to aluminum alloy specimens. This is further supported by the values of the hardening degree (U) as shown in Table 9.

As shown in Table 9, austenitic stainless steels exhibit significantly higher susceptibility to hardening when subjected to the innovative post-weld finishing method of weld bead removal. The machining of AISI 304L steel resulted in a substantial degree of hardening, reaching up to 136%. This phenomenon is directly associated with the surface finishing method, particularly the design of the multi-tooth cutting tool, which incorporates additional teeth at the end of the sequence to smoothen and burnish the machined surface. In contrast, the weld-joints made of aluminum alloys experienced a much lower degree of hardening. For the alloys EN AW-5083 H321 and EN AW-7075 T651, the degree of hardening was 47% and 57%, respectively.

## 4. Conclusions

The non-destructive inspection (PT, RT) revealed that the innovative post-weld finishing method enables the detection of welding defects, allowing for timely repairs during the production stage. This has the potential to enhance the reliability of welded structures and machine parts’ subassemblies.

The results revealed remarkably low surface roughness values for machining AISI 316L stainless steel, with Ra (Sa) = 0.295 (0.315) μm. Meanwhile, the surface topography testing after applying the innovative method to finish the weld of aluminum alloys EN AW-7075 T651 showed Ra (Sa) = 1.183 (1.184) μm, aligning with the expectations for a finishing treatment. Moreover, the analysis of the Abbott-Firestone curve demonstrated an almost “perfect” surface, with a bearing area of 60%, when finishing AISI 316L steel.

Metallographic testing revealed that welds made of stainless steel underwent greater plastic deformation than those made of aluminum alloys. The material of the AISI 316L steel weld deformed to a depth of approximately 6 µm, while the microstructure of the EN AW-7075 T651 aluminum alloy changed over an area of only 2.3 µm from the surface of the actual weld. Additionally, microhardness tests indicated that machining AISI 304L steel resulted in a significant degree of hardening, up to 136%. This phenomenon is directly related to the innovative post-weld finishing method, particularly the non-standard design of the multi-tooth cutting tool, which allows for the smoothing and burnishing of the machined surface.

We may conclude that the proposed innovative post-weld finishing method for the removal of the butt weld face of aluminum alloys and austenitic steels makes it possible to obtain surfaces of a very high quality. However, considering that the machined materials are unlikely to be protected from corrosion by paint coatings during operation, measures should be taken to prevent the formation of overgrowths on the cutting edges, for example, by increasing the cutting speed. This is crucial as the deep valleys that occur on the surface can become foci of fatigue or corrosion cracks in operational practice.

The future development of the presented topic will entail conducting research on the potential application of the innovative post-weld finishing method on fillet welded joints obtained through various techniques. Additionally, we will investigate the effect of this method on the corrosion resistance and reliability of the welded joint. Furthermore, we are currently involved in developing mathematical models to understand the relationship between surface roughness, hardness (degree of hardening), and several factors, including cutting conditions, cutting edge geometry, and material grade, during finishing.

## Figures and Tables

**Table 1:** The chemical composition of selected stainless steels.

Material	Chemical Composition [%]								
	C	Si	Mn	P	S	Cr	Ni	N	Mo
AISI 304L	0.021	0.425	1.340	0.0313	0.0056	18.214	8.106	0.0363	-
AISI 316L	0.023	0.390	0.850	0.0310	0.0021	16.700	10.180	0.0250	2.04

**Table 2:** The mechanical properties of selected stainless steels.

Material	R_m_ [MPa]	R_p0.2_ [MPa]	R_p1_ [MPa]	A50 [%]
AISI 304L	624	316	369	55.4
AISI 316L	583	268	316	55.0

**Table 3:** The chemical composition of selected aluminum alloys.

Material	Chemical Composition [%]								
	Si	Fe	Cu	Mn	Mg	Cr	Ni	Zn	Ti
EN AW-5083 H321	0.28	0.28	0.039	0.61	4.7	0.072	0.0057	0.15	0.018
EN AW-7075 T651	0.07	0.12	1.60	0.04	2.7	0.18	-	5.8	0.05

**Table 4:** The mechanical properties of selected aluminum alloys.

Material	R_m_ [MPa]	R_p0.2_ [MPa]	A50 [%]
EN AW-5083 H321	328 ÷ 336	251 ÷ 257	14 ÷ 15
EN AW-7075 T651	571 ÷ 572	493 ÷ 499	12 ÷ 13

**Table 5:** Results table of penetrant inspection for selected aluminum alloy samples after finishing.

Sample No.	Test Range	Indication No.	Location of Indications		
			X [mm]	Length [mm]	Breadth [mm]
5083	100%	1	2	1	1
7075	100%	1	5	2	2
		2	30	3	2
		3	52	2	2

**Table 6:** The 2D profile parameters’ measurement results.

Roughness Parameters [μm]	Specimen Material			
	AISI 304L	AISI 316L	EN AW-5083 H321	EN AW-7075 T651
Rq	1.646	0.497	2.355	1.952
Rt	13.875	6.110	17.644	20.046
Rz	6.888	2.560	9.268	8.360
Ra	1.158	0.295	1.636	1.183
Rc	4.278	1.524	5.935	6.569

**Table 7:** The 3D surface measurements results of the extracted area.

Roughness Parameters [μm]	Specimen Material			
	AISI 304L	AISI 316L	EN AW-5083 H321	EN AW-7075 T651
Sq	1.954	0.563	2.377	1.937
Sp	25.634	10.035	8.700	5.944
Sv	16.394	13.189	15.791	41.886
Sz	42.027	23.224	24.491	47.830
Sa	1.335	0.315	1.635	1.184

**Table 8:** Depth values of the deformation zone.

Sample Material	Depth of Deformation Zone [µm]	Standard Deviation
AISI 304L	3.1	1.0
AISI 316L	6.0	2.3
EN AW-5083 H321	2.7	0.8
EN AW-7075 T651	2.3	0.7

**Table 9:** Microhardness measurements and hardening degree results.

Sample Material	Microhardness		Hardening Degree (U) [%]
	After Finishing *HVp*	Before Finishing *HVs*	
AISI 304L	480	203	136
AISI 316L	457	200	129
EN AW-5083 H321	125	85	47
EN AW-7075 T651	163	104	57

## Data Availability

The data presented in this study are available on request from the corresponding author. The data are not publicly available due to privacy restrictions.
